# Supplementary material for: Bayesian refinement of protein structures and ensembles against SAXS data using molecular dynamics
Source: PLoS Comput Biol. 2017 Oct 18;13(10):e1005800. doi: 10.1371/journal.pcbi.1005800 (PMC5662244; doi:10.1371/journal.pcbi.1005800)
Supplement: S1 Archive — (BZ2) [file pcbi.1005800.s011.tar.bz2 › gromacs_4.62_bayesian_saxs_refi/share/html/online/g_polystat.html]

g\_polystat


| g\_polystat Main Table of Contents |

**VERSION 4.6  
Sat 19 Jan 2013**

---

### Description

g\_polystat plots static properties of polymers as a function of time
and prints the average.

By default it determines the average end-to-end distance and radii
of gyration of polymers. It asks for an index group and split this
into molecules. The end-to-end distance is then determined using
the first and the last atom in the index group for each molecules.
For the radius of gyration the total and the three principal components
for the average gyration tensor are written.
With option -v the eigenvectors are written.
With option -pc also the average eigenvalues of the individual
gyration tensors are written.
With option -i the mean square internal distances are
written.

With option -p the persistence length is determined.
The chosen index group should consist of atoms that are
consecutively bonded in the polymer mainchains.
The persistence length is then determined from the cosine of
the angles between bonds with an index difference that is even,
the odd pairs are not used, because straight polymer backbones
are usually all trans and therefore only every second bond aligns.
The persistence length is defined as number of bonds where
the average cos reaches a value of 1/e. This point is determined
by a linear interpolation of log(<cos>).

### Files

| option | filename | type | description |
| --- | --- | --- | --- |
| **-s** | topol.tpr | Input | Run input file: tpr tpb tpa |
| **-f** | traj.xtc | Input | Trajectory: xtc trr trj gro g96 pdb cpt |
| **-n** | index.ndx | Input, Opt. | Index file |
| **-o** | polystat.xvg | Output | xvgr/xmgr file |
| **-v** | polyvec.xvg | Output, Opt. | xvgr/xmgr file |
| **-p** | persist.xvg | Output, Opt. | xvgr/xmgr file |
| **-i** | intdist.xvg | Output, Opt. | xvgr/xmgr file |

### Other options

| option | type | default | description |
| --- | --- | --- | --- |
| **-[no]h** | bool | no | Print help info and quit || **-[no]version** | bool | no | Print version info and quit || **-nice** | int | 19 | Set the nicelevel || **-b** | time | 0 | First frame (ps) to read from trajectory || **-e** | time | 0 | Last frame (ps) to read from trajectory || **-dt** | time | 0 | Only use frame when t MOD dt = first time (ps) || **-tu** | enum | ps | Time unit: fs, ps, ns, us, ms or s || **-[no]w** | bool | no | View output .xvg, .xpm, .eps and .pdb files || **-xvg** | enum | xmgrace | xvg plot formatting: xmgrace, xmgr or none || **-[no]mw** | bool | yes | Use the mass weighting for radii of gyration || **-[no]pc** | bool | no | Plot average eigenvalues |

---

http://www.gromacs.org  
gromacs@gromacs.org
